# Supplementary material for: Learning Long Temporal Sequences in Spiking Networks by Multiplexing Neural Oscillations
Source: Front Comput Neurosci. 2020 Sep 7;14:78. doi: 10.3389/fncom.2020.00078 (PMC7505196; doi:10.3389/fncom.2020.00078)
Supplement: Supplementary file 1 [file Data_Sheet_1.PDF]

Supplementary Material

Network regime and error accumulation

We characterized the behavior of the balanced network by computing the average coefficient of variation (CV) and looking at fluctuations in the firing rate of the network. Fig.S1a shows that the neurons fire at relatively regular intervals (CV = 0.116), and Fig.S1b shows that the global activity is constant. The network is therefore in an asynchronous regular regime (Brunel, 2000). We tested the impact of adding jitter in the oscillation phase during training (see Fig. S8) on the performance during testing (without the phase jitter), and found that performance decreases as the noise increase during training.

Parameter exploration of the SRNN

Fig.S2 shows the impact of different network parameters on the model's performance. We used three measures of the network's activity and performance: 1- The average CV between trials for each cell. A low CV shows that the ratio of across-trial variability is low relative to the mean firing rate. 2- The average firing rate across all trials. 3- The average correlation between the output of the network and the target function after training Fig.S1d. We also investigated the impact of Gaussian noise added during training and testing, and found that the network is quite robust to the addition of this type of noise Fig.S1e. Even in cases where the input signal is buried in Gaussian noise, the network appears to be able to pick up on the signal and obtain decent performances.

Autonomous production of oscillatory activity

For a given network, it is possible to modulate the frequency of its activity by tuning the gain of the tonic input it receives. Stronger external inputs lead to a faster period, up to a limit after which the stability of the activity degrades (Fig.S3a,b). The activity of oscillatory networks remains stable in the presence of noise, as the network's cells received trains of synaptic inputs drawn from a Poisson distribution with increasing frequencies and the networks were all robust to clamping a subset of their neurons to their resting potential for the duration of the simulation (Fig.S3c).

We explored the impact of network parameters on the production of repeatable periodic activity. We were interested in networks that were spontaneously active and that switched to a synchronized regime when driven by a constant tonic input. Starting with networks identical to the main SRNN except for its size (500 cells instead of 1000), we tuned the gain and time-scale of inhibitory inputs as well as the gain of the external input and the network sparseness, and monitored the dynamics of the network. We found that all those parameters were crucial in shaping the transition between oscillatory and asynchronous regimes.

To assess to repeatability of network activity, we computed the Pearson correlation of the firing rate fluctuations during each trial with the first trial for every network (Fig.S4). Overall, regions of lower inhibition conductance and higher inhibitory synaptic time-constants favour the emergence of repeatable oscillations. The strength of the tonic input also had an impact on the network activity.

Finally, pairing the excitatory tonic inputs with inhibitory transients of variable durations allows us to control the relative phase of the oscillators. However, this feature might be a concern if the networks are too sensitive to the exact timing of this input. We performed a sensitivity analysis in Fig. S3d, by adding some jitter to the end of each inhibitory transient drawn from a normal distribution $N(0, \sigma)$, where σ is represented on the x-axis.

Rescaling slopes

We examined whether the qualitative differences in performance slopes between faster and slower rescaling is reflected in the SRNN's activity. We first obtained the projection of the SRNN's activity on the first principal component (accounting for most of the variance of the SRNN, which is typical for this type of network (Aljadeff et al., 2016)). We then scaled back the activity of the first principal component to the original scaling for all the rescaling factors tested (Fig.S7a). This allowed us to compare the features of the SRNN's activity at the original scale. We used a Fourier analysis to obtain a representation of the SRNN's activity for all rescaling factors at different frequencies (Fig.S7b). For each rescaling and each frequency band tested, we computed an "error" value defined as the mean squared difference between the SRNN's activity scaled back to the original velocity and the activity of the SRNN on the original scaling at a given frequency. Each error value was then normalized by the square of the frequency band for the target (thus making comparisons of error across frequencies possible).

This analysis revealed that the higher frequencies of the network activity deteriorated faster than the lower frequencies. The lower frequencies were well-preserved for inputs that were slowed down, but were progressively lost for faster rescalings. This explains why the performance reached a plateau (in the absence of input noise) for longer rescaling. We used the same normalization process to examine the output performance across different velocities (Fig.S7c). We computed the error for each frequency until 6 Hz given that the target function was generated by applying a low-pass filter on white noise at this cut-off. All frequencies degraded evenly for faster velocities. However, unlike the SRNN's activity, the higher frequencies appeared to be more preserved for the output, whereas the lower frequencies (<4Hz) were more degraded. These results show that rescaling the input frequencies lead to complex modifications of the SRNN dynamics, and highlight a non-trivial relationship between the input and output of the model.

Input oscillations and phase jitter

We simulated a random-walk process $\phi(t)$ (see Methods) with a standard deviation σ_ϕ that we added to the phase of an oscillator (Fig.S8a). Because the noise of the random walk comes from a Gaussian process with mean 0, the expected deviation from the base sinusoid is null at all time steps, but its standard deviation at time t is equal to $\sqrt{t\sigma_\phi^2}$ (Fig.S8b).

We then trained our model with different σ_ϕ values for different durations. Fig.S8c shows the expected standard deviation in degrees for the σ_ϕ used in our simulation, and well as the corresponding error between the output and target functions. Our results show that error accumulates exponentially (R^2 = 0.92, Fig.S8d). This means that networks can tolerate a deviation of up to 50 degrees from the original sine wave before reaching random performance (defined as the plateau in MSE of the output in Fig.S8c,d).

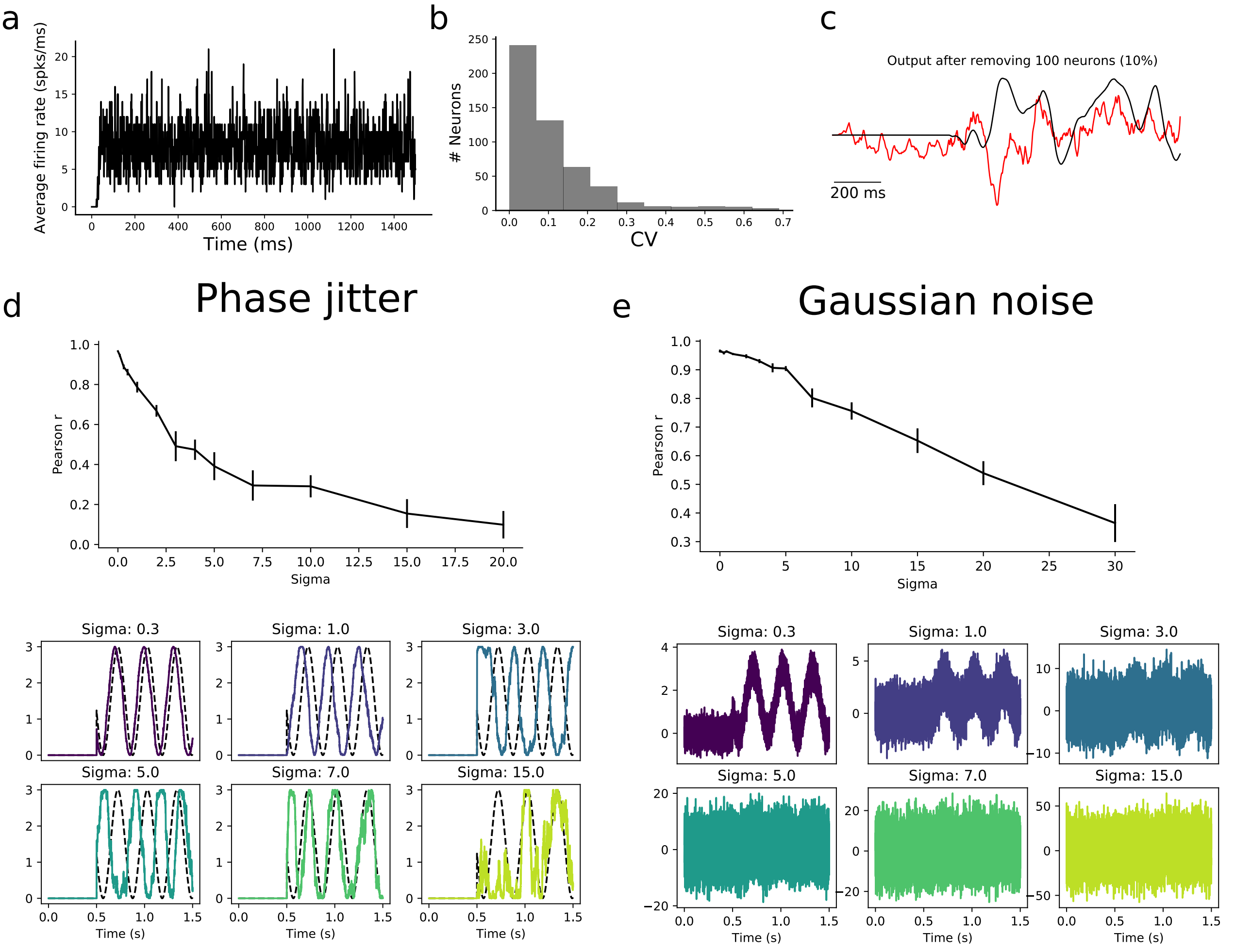

Figure S1. Network regime and error accumulation. **a** Number of spikes per ms across the network shown in Fig.1. **b** Histogram of the CV for each neurons of the network. The average CV is 0.116. **c** Output (red) of a network after removing 10% of its neurons. **d** Impact of phase jitter added during training on testing performance. The bottom of the panel shows samples of input oscillation at different sigma of phase jitter. (e) Impact of Gaussian noise applied during training and testing, with input samples at the bottom of the panel.

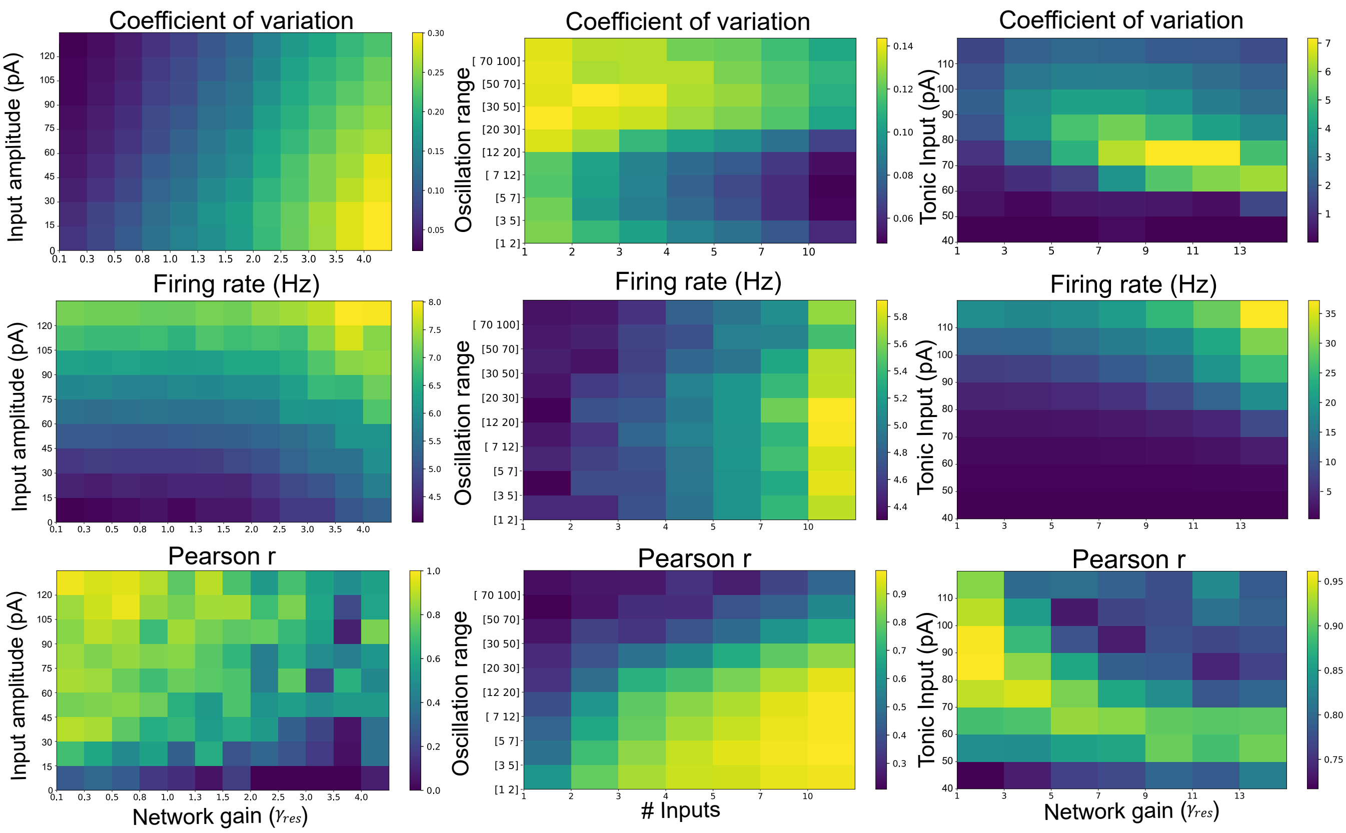

Figure S2. Parameter exploration for training a SRNN. The top row shows the average CV, the middle row shows the average firing rate, and the bottom row shows the average correlation between the output of the network and the target output. *Input amplitude* is the strength of the the input projecting to the SRNN's units. *Network gain* is the strength of the recurrent connections between the SRNN's units. *Oscillation range* is the range from which each input unit's sine wave period is randomly drawn. *# Inputs* is the number of different input units projecting to the SRNN. *Tonic input* is the strength of the constant current injected to each of the SRNN's units.

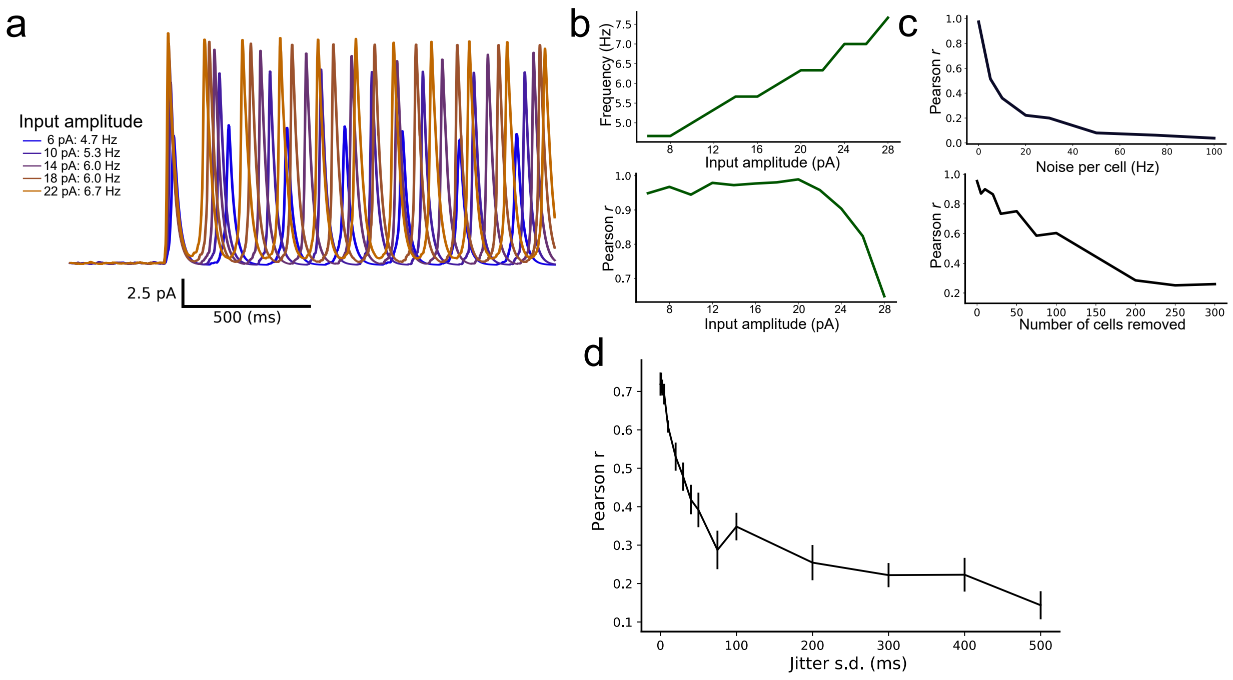

Figure S3. Autonomous production of repeatable periodic activity. **a** Average current over all neurons for five trials of each condition as a function of the strength of the external drive. The frequency scales with the strength of the tonic input. **b** Top: Average frequency of the network's activity as a function of the external drive. Bottom: Pearson correlation between multiple trials as a function of the amplitude of the external drive. **c** Pearson correlation between multiple trials for a given network as a function of the rate of noisy synaptic input per neuron (top) and as a function of the proportion of clamped neurons (bottom). **d** Sensitivity analysis for the precision of the tonic input timing to the oscillatory networks on the network performance.

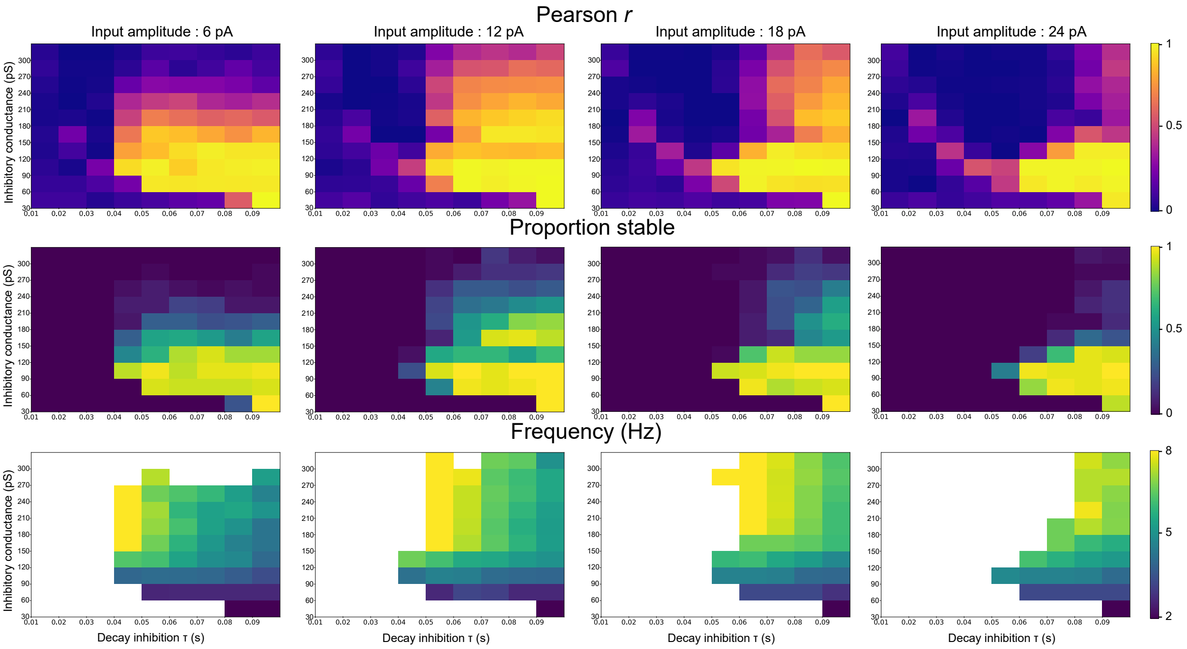

Figure S4. Parameter exploration of the network's synchronous activity (inhibition conductance and kinetics). Each column represents a different strength of the external input step, and each heatmap shows different combinations of the conductance and synaptic time-constant of inhibitory inputs. The top row shows the average Pearson correlation between the output and the target function for 5 trials for each of the 50 random network initializations tested per condition. The middle row shows which proportion of the networks are considered as stable. The bottom row shows the average frequency of the periodic activity of the stable networks.

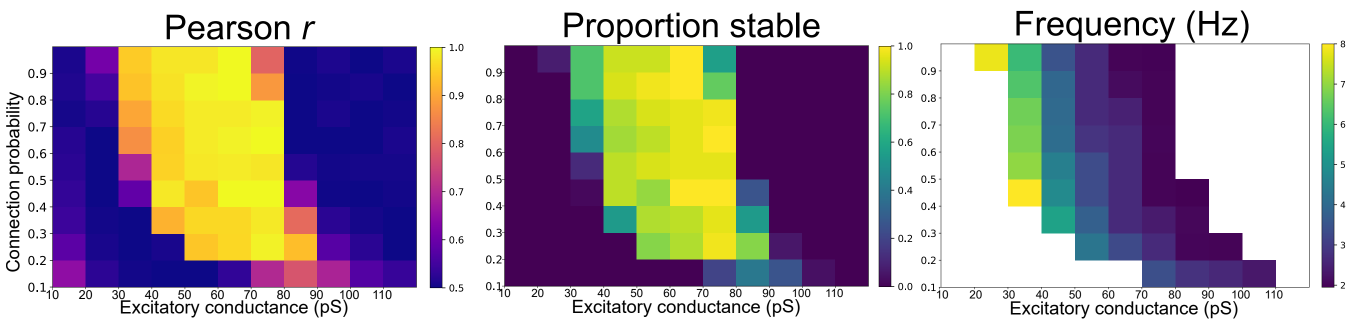

Figure S5. Parameter exploration of synchronous activity (excitatory conductance and p). The sparseness of the SRNN and the conductance of the excitatory connections were systematically varied to observe their impact on the network's regime. *Left*: Pearson correlation between the output and the target function for 5 trials for each of the 50 random network initializations tested per condition. *Middle*: proportion of networks that are considered stable. *Right*: average frequency of the periodic activity of the stable networks.

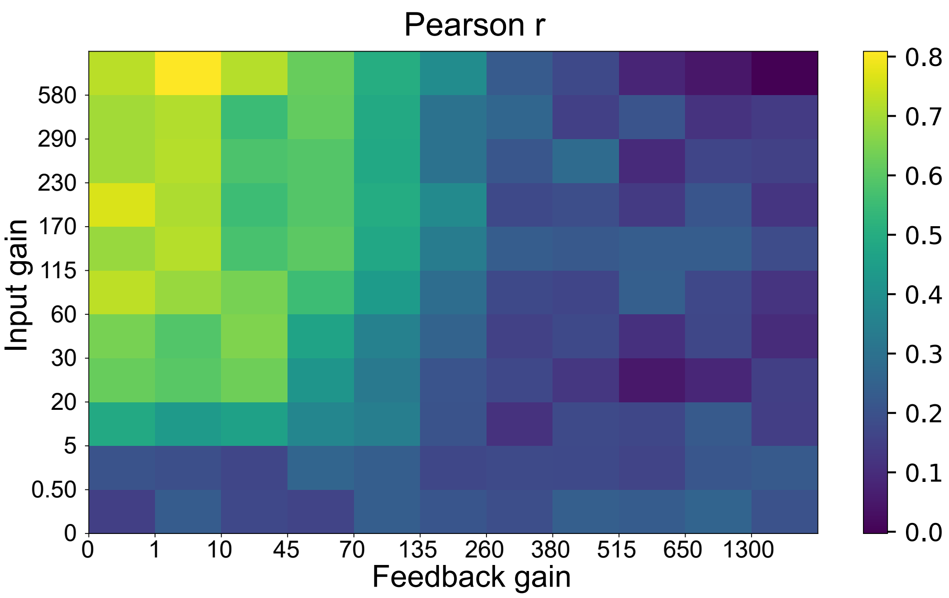

Figure S6. Impact of SRNN feedback to the oscillatory networks. The models can perform well when the gain of the feedback connections from the SRNN to the oscillatory networks are scaled up to a limit. The effect of the feedback gain appears to be independent of the strength of the tonic input of the step function to the oscillatory networks.

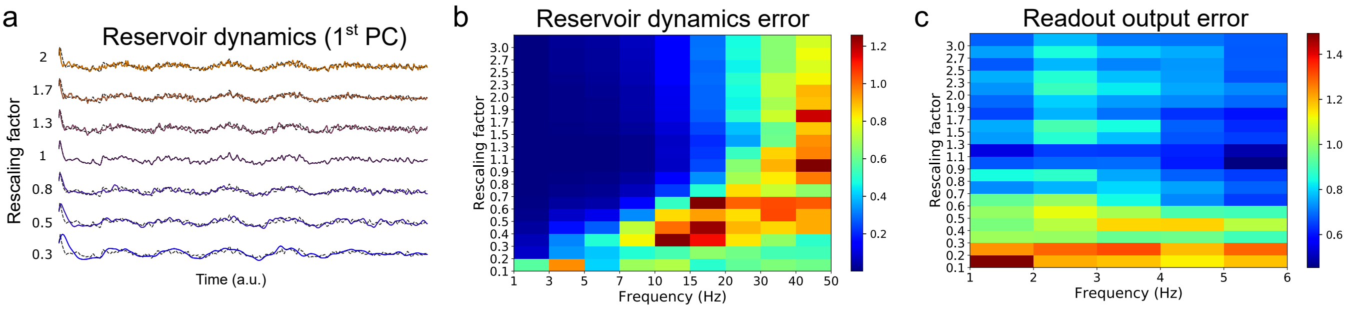

Figure S7. Network dynamics and performance with temporal rescaling. **a** Activity of the SRNN projected on the first principal component for different rescaling factors. The black dashed line represents the activity without rescaling. The traces displayed have been scaled back to the original velocity. **b** Normalised error of the first principal component of the SRNN's activity for different rescaling factors and frequency bands. **c** Normalised error of the network's output (MSE between the output and the target divided by the total variance of the target function) for different rescaling factors and frequency bands.

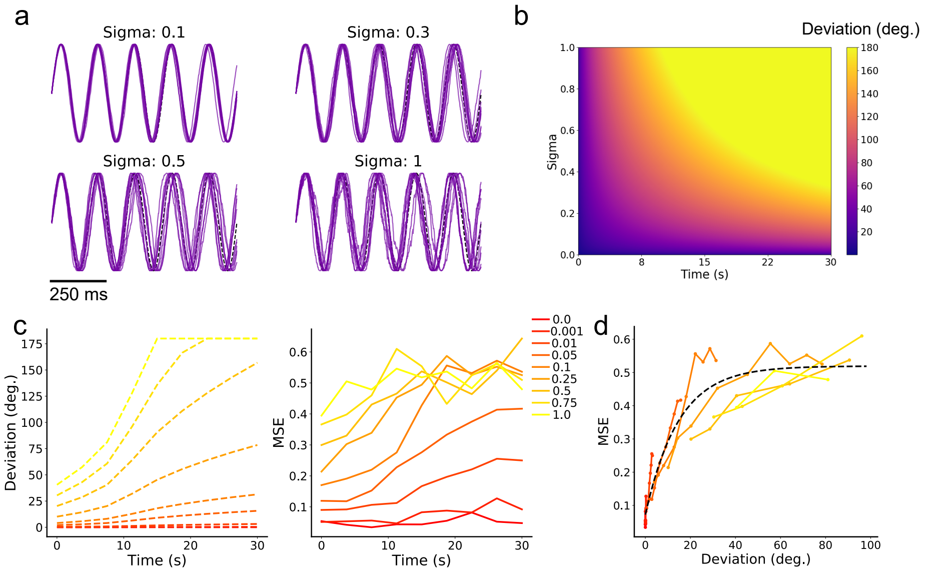

Figure S8. Network performance with desynchronized inputs. **a** Four simulations of 10 trials each with different σ_ϕ with a random walk process added to the phase of a sine wave. **b** Analytical standard deviation (in degrees) around the expected phase of a deterministic signal as a function of time at different values of σ_ϕ. **c** Left: Analytical estimation of the standard deviation in degrees with the different σ_ϕ used for the network simulation. Right: corresponding MSE between the output and target of the readout unit for the different conditions tested. **d** Relationship between the deviation in degree and the MSE. An exponential function was fit to the data (R^2=0.92).

REFERENCES

Aljadeff, J., Renfrew, D., Vegué, M., and Sharpee, T. O. (2016). Low-dimensional dynamics of structured random networks. *Physical Review E* 93, 022302. doi:10.1103/PhysRevE.93.022302

Brunel, N. (2000). Dynamics of sparsely connected networks of excitatory and inhibitory spiking neurons. *Journal of computational neuroscience* 8, 183–208
